# An exploratory study of what happens to women who are denied abortions in Cape Town, South Africa

**DOI:** 10.1186/s12978-015-0014-y

**Published:** 2015-03-21

**Authors:** Jane Harries, Caitlin Gerdts, Mariette Momberg, Diana Greene Foster

**Affiliations:** Women’s Health Research Unit, School of Public Health and Family Medicine, Faculty of Health Sciences, University of Cape Town, Anzio Road, Observatory, 7925 Cape Town South Africa; Advancing New Standards in Reproductive Health, University of California, San Francisco, 1330 Broadway St, Suite 1100, Oakland, CA 94612 USA

## Abstract

**Background:**

Despite the change in legal status of abortion in South Africa in 1996, barriers to access remain. Stigma associated with abortion provision and care, privacy concerns, and negative provider attitudes often discourage women from seeking legal abortion services and sometimes force women outside of the legal system.

What happens when women present for abortion at a designated abortion facility and are denied abortions due to gestational limits or other factors–is unknown. Whether women seek care at referral facilities, seek illegal abortion, or carry pregnancies to term has never been documented. This study, part of a multi-country Global Turnaway Study, explored the experiences of women after denial of legal abortion services.

**Methods:**

Qualitative research methods were used to collect data at two non-governmental organization health care facilities providing abortion services. In depth interviews were held with women 2 to 3 months after they were denied an abortion. Data were analyzed using a thematic analysis approach.

**Results:**

The most common reason for being turned away was due to gestational age over 12 weeks with some women denied abortions that day because they did not have enough money to pay for the procedure. Almost all women were extremely upset at being denied an abortion on the day that they visited the health care facility. Some women were so distressed that they openly discussed the option of seeking an illegal provider or exploring the possibility of securing another health care professional who would assist them.

**Conclusions:**

Despite South Africa’s liberal abortion law and the relatively widespread availability of abortion services in urban settings, women in South Africa are denied abortion services largely due to being beyond the legal limits to obtain an abortion. A high proportion of women who were initially denied an abortion at legal facilities went on to seek options for pregnancy termination outside of the legal system through internet searches--some of which could have led to unsafe abortion practices. Further efforts should be directed towards informing women in all communities about the availability of free services in the public sector and educating them about the dangers of unsafe methods of pregnancy termination.

## Background

The South African Choice on Termination of Pregnancy (CTOP) Act, passed in 1996, replaced the previously restrictive Abortion and Sterilization Act of 1975. The CTOP Act provides for abortion on request up to and including 12 weeks of gestation, thus promoting a woman’s reproductive right to have an early, safe and legal abortion. In cases of socio-economic hardship, rape, incest and for reasons related to the health of the pregnant woman or fetus, terminations can also be performed up to 20 weeks of gestation. From 20 weeks onward terminations are available under very limited circumstances. As a result of this legislation, abortion-related morbidity and mortality were estimated to have declined by 91.1% [1]. Despite the change in legal status of abortion, however, barriers to access remain. These include provider opposition, stigma associated with abortion, poor knowledge of abortion legislation, a lack of providers trained to perform abortions and facilities designated to provide abortion services notably in the rural areas [2-6]. Because many facilities do not have doctors on staff who are willing or able to perform second trimester procedures, women are often denied abortions where they seek care and referred to other facilities or asked to return on a pre-scheduled day.

A small body of research exists that explores barriers to abortion access after the 1996 law change. Some studies have reported that effects of stigma, privacy concerns, and conscientious objection by providers often discourage women from seeking abortion services within the public sector and sometimes force women outside of the legal system entirely [5,7]. Two recent studies shed some light on reasons why women access abortions outside of designated abortion facilities and/or attempt self-induction prior to seeking a legal abortion [2,6]. One study found that among women seeking second-trimester abortions in South Africa, 17.5% reported a failed attempt at self-induction prior to seeking legal abortion services [2]. The second study found that among women presenting to hospitals with incomplete miscarriages, nearly half had attempted self-induction and another quarter of women had sought services from a traditional healer [6]. More than half of women in that study did not know the legal status of abortion in South Africa, and among those who were aware of the law, 17% had been deterred from seeking legal services due to anticipated fear of rude medical staff and expectations of poor service quality.

More recently concerns have been raised in the media and within the South African Health Department about an increasing proliferation of illegal, unlicensed providers who pose as legal abortion providers via the internet and by advertising in public spaces [8].

What happens when women present for abortion at a designated abortion facility and are denied abortions due to gestational limits or other factors is unknown. Whether women seek care at referral facilities, seek illegal abortion, or carry pregnancies to term has never been documented. How women learn about and seek illegal abortions in South Africa, and how legal abortion, illegal abortion and unwanted childbirth affect women’s health and well-being are questions that remain unanswered. This study conducted in Cape Town, South Africa formed part of the Global Turnaway study. The Global Turnaway study, a pilot feasibility study conducted in five countries where abortion is legal: Bangladesh, Colombia, Nepal, Tunisia, and South Africa, aimed to understand the experiences of women after denial of legal abortion services and explore subsequent decision making after being denied an abortion. These data can help to inform the development of programs and policies to improve access to and utilization of safe abortion services in South Africa and elsewhere [9,10].

## Methods

### Study sites

The study was conducted in Cape Town, South Africa over three months in 2013 at two non-governmental organization (NGO) health care facilities providing abortion services. NGO facilities were selected as they provide abortion services on a daily basis and due to logistical reasons. Women who were in the second trimester were also often referred from public sector facilities due to provider shortages and thus representative of some women accessing the public sector. Second trimester services were provided once or twice per week dependent on provider availability. Women who were more than 12 weeks pregnant were required to return to the facility on a pre-scheduled day.

### Data collection

Two research assistants trained in qualitative research methods, approached women who were denied an abortion on the day they sought an abortion and asked whether they would agree to be contacted in 2 months’ time to discuss their current situation. Information obtained at the baseline interview included preference for mode of future contact (phone versus in person), contact details and reasons for being turned away on the day they sought an abortion. Clinic staff assisted study staff in identifying women who had been turned away on the day they sought an abortion.

Contact details were obtained after the woman had given consent to be contacted by the research team. Women who consented were invited to participate in a semi-structured in-depth interview two months following being turned away on the day they sought an abortion. The aim of these interviews was to discuss subsequent decision making processes after being denied an abortion and to assess knowledge and use of methods of illegal abortion. Socio demographic information including age, marital status, reproductive history and employment status was collected prior to the in- depth interview. The mode of interview (telephone or in-person) and confidentiality preferences were agreed upon at baseline. Text-messages or e mail reminders were sent prior to the follow-up interview where appropriate. If after at least 5 reminder and contact attempts women were not available, no further contact was initiated. Women were given compensation of ZAR 50 at the baseline interview for time spent providing information, and ZAR 100 as airtime or in cash, according to women’s preferences for the lengthier in- depth interview.

Eighteen women were denied an abortion on the day they sought an abortion. Of these 18 women, 2 refused participation due to time constraints and one woman was under 18 years of age and thus not eligible. A total of 15 women consented to participate in the study. Of the 15 who consented and were eligible to participate, we were able to follow-up 8 of the 15 women and unable, despite repeated attempts, to contact the remaining 7 women. A retrospective review of facility records indicated that 2 of the 7 women the research team were unable to follow- up had returned for an abortion. All interviews were by telephone apart from one face to face interview which was conducted in a neutral space. Contacting women 2- 3 months after being denied an abortion proved to be challenging and required repeated attempts at follow-up. Contact details changed or women did not respond to repeated reminder calls. Careful record keeping was maintained around number of attempts used to contact participants.

The research instrument in the form of an interview guide was open-ended, and included probes for potential additional issues that could emerge as important concerns. Some of the key themes explored included; reasons and subsequent response to being denied an abortion; decision making process once denied an abortion and choice of places including self-induction or unlicensed abortion providers.

### Data analysis

Conventional thematic analysis was conducted on the interview data to identify key themes. Initial categories for analyzing data were drawn from the interview guide, and then themes and patterns were identified after reviewing the data. The categories in the interview guide were linked to the key research question i.e. what happens to women who are turned away after seeking a legal abortion, do they continue with the pregnancy or seek assistance elsewhere including illegal providers. Due to the small sample size all data was manually coded by the first author.

### Ethical considerations

Ethical approval to conduct the study was obtained from the Human Research Ethics Committee, University of Cape Town. All study participants provided written informed consent prior to the interview process. Verbal permission was obtained prior to digitally recording all interviews. Confidentiality and anonymity was ensured. Participants were assured that in all forms of dissemination, including publications and dissemination meetings, participants would not be identified by name, facility or any other identifier. All data were closely controlled and stored in locked files and password protected computer files. Digital recordings were erased once they had been cross checked after data transcription.

## Results

### Socio- demographic characteristics

Socio-demographic characteristics were collected from all 8 women who were followed up. The mean age was 27 years (range 20-34), all women were employed, had finished high school and had at least one child with 5 out of 8 women stating they had used a contraceptive method prior to falling pregnant. Reasons for being turned away included beyond the legal limit (2 women); in the second trimester and insufficient funds (4 women) and too early to determine gestational age (2 women).

### Pregnancy outcomes of women turned away

Of the 8 women interviewed, 5 returned to the health care facility and obtained an abortion, of the remaining 3, 2 decided to continue with the pregnancy and the third women, despite being beyond the legal limit, obtained an abortion elsewhere. It was unclear exactly where she obtained an abortion as she was reluctant to expand further than saying it was a “health care clinic”. Of the 7 women who we were not able to follow up, 2 returned to the abortion facility verified by clinic records. Information about the remaining 5 women whom we were unable to contact is unknown.

### Reasons for not receiving an abortion

Reasons for being denied an abortion ranged from too early in the pregnancy to determine gestational age (GA) to beyond the legal gestational age limit i.e. greater than 20 weeks. In some instances, women who were beyond 12 weeks gestational age were asked to return on a pre-scheduled day when a doctor was available to perform a second trimester abortion. There were two instances where women did not have enough money to pay for a second trimester procedure on the day, and had to return to the clinic one week later once they had obtained the requisite amount of money to pay for the procedure.

### Response to being turned away on day of recruitment

Almost all women were visibly upset and distressed on being denied an abortion on the day that they visited the health care facility. For those women who were beyond the legal limit to obtain an abortion it was particularly traumatic. Some women were so distressed that they openly discussed the option of seeking an illegal provider or exploring the possibility of securing another health care professional who would assist them. In addition, some women wanted to make certain that the gestational age calculation was accurate and requested a repeat ultrasound to confirm that the reading was indeed correct.

A woman who was denied an abortion as she was beyond the legal limit (22 weeks) did not realize she was pregnant until a routine doctor’s visit and was both angry and desperate as this extract illustrates:

*I didn’t know that I was pregnant until yesterday as I was taking birth control. I had a baby a few months ago and went onto the pill … I didn’t get my period after going onto the pill and the pharmacist told me that it was normal not to get a period for up to 4 months after having a baby. I only found out because I went in for a gynecologist appointment and the doctor felt my stomach and said “Are you pregnant?”*

*There must be someone who can help me. They do it up to 24 weeks in the UK… The government is basically forcing me to get a backstreet abortion.*

At the subsequent interview 8 weeks later she was adamant that she had not been 22 weeks pregnant at the time and thus incorrectly denied an abortion. She recounted how she went to another “private doctor” who informed her she was 11 weeks pregnant, not 22 weeks, as the clinic had indicated. She was given “pills”, but could not recall how many, nor the name of the pills and subsequently indicated that she had undergone a procedure in a government clinic. She was reluctant to provide further details stating it was a “private matter and would rather not say”, suffice to say she had been assisted and was no longer pregnant and was back at work. A subsequent retrospective review of facility records confirmed the gestational age by ultrasound at 22 weeks.

Distress and frustration were further enhanced for two women who had travelled a great distance to secure an abortion, including from a neighboring country (Namibia) where abortion is restricted and from a city more than 1400 kilometers away to ensure privacy and anonymity, explained by a woman who had travelled from Johannesburg: “You know the chances of me bumping into someone is so huge that I would rather not take that risk and rather take a flight to Cape Town to go and do it there”.

Similarly, for those women who had to return when a doctor was available found having to wait challenging. Once a decision was made extending the time period exacerbated the decision making process. A woman described how she “just wanted the procedure over and done with as she had made peace with her decision”.

All women were fairly clear in their reasons for wanting to terminate an unplanned pregnancy and indicated they were not currently able to have a child based on their personal and socio-economic circumstances including reaching their desired family size.

### Decision making processes and actions after being turned away

We asked women who had been denied an abortion whether they had considered obtaining an abortion elsewhere. Out of the 8 women who had been turned away, 3 had accessed an illegal provider but did not follow through with an illegal abortion; they either returned to the clinic or continued with the pregnancy. Women spoke about undertaking google searches for abortion clinics. In their searches, women described being confronted with a host of abortion clinics advertising “quick results” including postal delivery of tablets. Although these online clinics pose as “legal, safe abortion clinics”, most women realized that they were illegal providers after making initial telephonic contact or, in two reported instances, visiting an unlicensed abortion provider. Of the women who contacted illegal providers in this fashion, none of them went through with the process of obtaining an abortion from any of the providers they contacted.

Three out of the eight women turned away expressly recounted their experiences with these unlicensed abortion providers posing as legal providers. A woman who was beyond the legal gestational limit explained:

*I phoned one of these clinics that were advertising abortions online The person said that they will come down to you and then they will like give you the pills and stuff, but I asked them – you’re not going to any clinic or something where they can actually see if you’re okay? They said – no, it is very safe but I decided against it. They wanted to meet me somewhere in town and charge R 750 for the pills but I decided it wasn’t right as there was no clinic or anything else.*

This woman subsequently decided to continue with her pregnancy and planned to be sterilized postpartum.

A woman who was still within the legal limits recounted a disturbing experience of visiting an illegal provider via an advert on the internet. Her motivation for seeking assistance from an “illegal provider” was based on cost and not wanting to access the public sector due to past negative experiences. She recounted in lengthy detail her experience of visiting an illegal abortion provider in central Cape Town. She did subsequently return to the study facility for an abortion.

It is important to note that she was well informed about the law yet still made contact with an illegal abortion provider knowing it was not a safe, legal abortion clinic.

*I was extremely desperate. I googled everything from abortion clinics on the internet, everything from that abortion drug until I found someone because they were offering to drop it off. … If you google abortion clinic sites, .. You will find hundreds of them, and to be honest, what also scared me was going to one of our public hospitals…*

*I decided to try one of these numbers and he called back immediately and I said –I want to make an appointment – I explained to him that I was thinking of maybe coming to have a look… He said – there’s an ATM on the corner, I’ll meet you there. And every little bit of common sense that I had was saying – no you won’t… I’m not going to do anything, OK let me just see, … I met him and there’s like a cell phone shop .. you could walk through and then there was a trailer. There was a lopsided bed… I asked him – but you advertised a clinic, there is no clinic… I asked how this works. He said –the pills are R1 200 … he didn’t even speak about any kind of examination. He didn’t even ask how far pregnant I was … I realized it was not a real clinic, because I know that at Marie Stopes until a certain point you could go it’s regulated and there is a clear time table,. I asked where’s the after-care clinic. And then he says it’s very, very safe. … he could help insert the four tablets into your woman’s parts … I had to either stop fooling myself into thinking that something’s going to happen and I knew that this is as far as it goes and I would have to actually go and seek help at a government facility or something and I resigned myself to that and I left…*

Whilst some women who were turned away sought illegal providers another woman who had to return for a second trimester procedure decided to continue with the pregnancy after viewing the ultrasound image.

*I asked the nurse if could see the baby and he asked me twice if I was really sure if I wanted to see the baby … and then I was able to see on the ultrasound scan. I think it’s just a thing that once you see that baby on the screen and it’s kicking, I just told him – you know, I can’t do this.*

A married woman with three small children who was beyond the legal limit (22 weeks) decided to continue with the pregnancy after discussion with her husband. Initially on being turned away she recounted how she had “considered other options” despite being aware of the health risks involved in seeking an illegal provider.

*I did consider backstreet - I considered going somewhere else. I googled abortion providers and made telephonic contact and was told it would cost R 750 for pills but I had to meet the person to get the pills…and that’s very dangerous and risky so I decided to find out some more. I telephoned the number and the person they will come down to you and then they will like give you the pills, but I asked them – you’re not going to any clinic or something where they can actually see if you’re okay … He said – no, it’s very safe but I decided against it.*

The above excerpts indicate that despite being aware of the dangers of backstreet providers and the requirements for a legal abortion, women still sought out these options. Moreover, some of the women had decided against visiting public sector facilities due to past negative experiences particularly in the maternity setting or with family members who had not received adequate care.

## Discussion

Women’s responses to being denied an abortion varied, some were able to return at a later date, others decided to continue with the pregnancy and yet others who were denied an abortion did seek out illegal abortion providers though none actually followed through with the service. Women at the study sites were denied an abortion on legal grounds largely due to advanced gestational age. In some cases, though women sought abortions within the legal limits, they were required to return at a later stage when a second trimester provider was available. This highlights the shortage of trained second trimester abortion providers underscored by a relatively high proportion of second trimester abortions in South Africa. Approximately 25% of abortions in South Africa are performed after 12 weeks whereas in most developed countries approximately 10% of abortions are in the second trimester [11].

Overall women were aware of the legal status of abortion in South Africa, including gestational age restrictions. Nevertheless, most women who were denied an abortion did make contact with illegal providers. Negative perceptions and experiences of public sector abortion services has been previously reported in South Africa [6,12,13], however, this is the first study where women have knowingly accessed illegal providers due to prior dissatisfaction with public sector abortion facilities and highlights the need to improve abortion services and access at public facilities. Our data suggest that illegal providers, both online and in person, are dispensing misoprostol for pregnancy termination. Similar to a previous study conducted in the same geographical area, our study suggests that invasive methods including attempts at instrumentation of the cervix is not taking place [2]. What medication protocols (if any) are recommended or whether assessment of gestational age or other contraindications for medical abortion is undertaken by such providers is unknown, and warrants further investigation.

The proliferation of unlicensed abortion providers located in close proximity to legal abortion clinics is of concern and is reflective of the fact that such providers are in demand and able to operate openly in public spaces. In our study, women’s experiences with illegal providers ultimately discouraged them from obtaining abortions from such providers. However, because we recruited participants for our study from legal abortion facilities, it is possible that our sample might have been predisposed to seeking legal means of pregnancy termination. The results of this study may not reflect women who do not present at legal abortion facilities but go directly to illegal, unlicensed providers, or who successfully self-induce abortion outside of legal facilities. More research to identify women who successfully terminate pregnancies outside of the legal system would help to improve understanding of the barriers that women face in seeking legal services in South Africa.

This study had several limitations. The research was conducted in a predominantly urban area with numerous public and private abortion facilities and may not be generalizable to other areas within South Africa. One would expect that in areas with fewer legal abortion facilities accessing illegal providers might be even higher yet this was not necessarily the case. Another possible limitation to this study is that we did not recruit women from public sector facilities but from fee paying abortion clinics. Despite our sample's relative higher socioeconomic status than women accessing public sector abortion services, the women in our study still made attempts to access illegal abortion services.

## Conclusions

Despite South Africa’s liberal abortion law and the relatively widespread availability of abortion services in urban settings, barriers to abortion access remain. Despite the small sample size in our study, a high proportion of women who were initially denied an abortion at legal facilities went on to seek options for pregnancy termination outside of the legal system--some of which could indeed have led to unsafe abortion practices. To address barriers to abortion access in South Africa, abortion provision in public health facilities must be strengthened and improved. Data from our study reinforces evidence of a negative perception of abortion services at public facilities similar to previous studies conducted in South Africa [5,6,13,14]. The South African Health Department should work to destigmatize abortion within the health system, quality of service provision must be improved, and availability of services must be strengthened. Additionally, further efforts should be directed towards informing women in all communities about the availability of free services in the public sector and educating them about the dangers of unsafe methods of pregnancy termination.
